# Metabolic targeting of HIF-dependent glycolysis reduces lactate, increases oxygen consumption and enhances response to high-dose single-fraction radiotherapy in hypoxic solid tumors

**DOI:** 10.1186/s12885-017-3402-6

**Published:** 2017-06-15

**Authors:** Eric Leung, Rob A. Cairns, Naz Chaudary, Ravi N. Vellanki, Tuula Kalliomaki, Eduardo H. Moriyama, Hilda Mujcic, Brian C. Wilson, Bradly G. Wouters, Richard Hill, Michael Milosevic

**Affiliations:** 1 Department of Radiation Oncology, Sunnybrook Health Sciences Centre and Odette Cancer Centre, Toronto, Canada; 2 Radiation Medicine Program, Princess Margaret Cancer Centre, Toronto, Canada; 3 Ontario Cancer Institute, Princess Margaret Cancer Centre, University Health Network, Toronto, Canada; 4 Department of Radiation Oncology, University of Toronto, Toronto, Canada; 5 Department of Medical Biophysics, University of Toronto, Toronto, Canada

**Keywords:** Hypoxia, Hif-1α, Glycolysis, Lactate, Radiation response, Metabolism, High-dose single-fraction radiation

## Abstract

**Background:**

A high rate of glycolysis leading to elevated lactate content has been linked to poor clinical outcomes in patients with head and neck and cervical cancer treated with radiotherapy. Although the biological explanation for this relationship between lactate and treatment response remains unclear, there is a continued interest in evaluating strategies of targeting metabolism to enhance the effectiveness of radiotherapy. The goal of this study was to investigate the effect of metabolic-targeting through HIF-1α inhibition and the associated changes in glycolysis, oxygen consumption and response on the efficacy of high-dose single-fraction radiotherapy (HD-SFRT).

**Methods:**

HIF-1α wild-type and HIF-1α knockdown FaDu and ME180 xenograft tumors were grown in the hind leg of mice that were placed in an environmental chamber and exposed to different oxygen conditions (air-breathing and hypoxia). Ex vivo bioluminescence microscopy was used to measure lactate and ATP levels and the hypoxic fraction was measured using EF5 immunohistochemical staining. The oxygen consumption rate (OCR) in each cell line in response to in vitro hypoxia was measured using an extracellular flux analyzer. Tumor growth delay in vivo was measured following HD-SFRT irradiation of 20 Gy.

**Results:**

Targeting HIF-1α reduced lactate content, and increased both oxygen consumption and hypoxic fraction in these tumors after exposure to short-term continuous hypoxia. Tumors with intact HIF-1α subjected to HD-SFRT immediately following hypoxia exposure were less responsive to treatment than tumors without functional HIF-1α, and tumors irradiated under air breathing conditions regardless of HIF-1α status.

**Conclusions:**

Blocking the HIF1 response during transient hypoxic stress increased hypoxia, reduced lactate levels and enhanced response to HD-SFRT. This strategy of combining hypofractionated radiotherapy with metabolic reprogramming to inhibit anaerobic metabolism may increase the efficacy of HD-SFRT through increased oxygen consumption and complementary killing of radiosensitive and hypoxic, radioresistant cells.

**Electronic supplementary material:**

The online version of this article (doi:10.1186/s12885-017-3402-6) contains supplementary material, which is available to authorized users.

## Background

It has been estimated that over 60% of tumors predominately utilize glycolysis for energy production and survival [1]. This shift to a glycolytic metabolism from oxidative phosphorylation was first described eighty years ago by Warburg who observed that cancer cells have high rates of glycolysis even in the presence of high oxygen [2]. In a low oxygen environment, there is an adaptive decrease in mitochondrial respiration resulting in high glycolysis described as the ‘Pasteur Effect’. Like the ‘Warburg Effect’, this phenomenon is also observed in malignant cells since many tumors are hypoxic, due in part to unregulated angiogenesis and the development of abnormal vasculature.

A high rate of glycolysis leading to high tumor lactate concentration has been linked to poor clinical outcomes in patients with head and neck or cervical cancer treated with fractionated radiotherapy [3, 4] and impaired response to radiation treatment in pre-clinical studies [5]. Interestingly, Quennet et al. demonstrated an inverse correlation between lactate content and the radiation response of head and neck xenografts that was largely independent of tumor hypoxia [5]. The biological explanation for this relationship between lactate and treatment response is not fully understood but mounting evidence suggests that it is a multifactorial effect of hypoxia, altered metabolism and the inherent biological aggressiveness of certain tumor types [6]. Studies have also found that lactate itself may directly affect radiation response through free radical scavenging by pyruvate [7, 8]. Taken together, these observations suggest that inhibiting glycolysis and lactate production may sensitize tumors to radiotherapy and improve clinical outcomes in patients.

Hypoxia inducible factor 1α (HIF-1α) is an important determinant of the switch to anaerobic metabolism in both normoxic and hypoxic tumor cells [9]. Increased HIF-1α signaling due to hypoxia, altered gene expression or the direct effects of radiation [9–11] is associated with upregulation of pathways involved in glycolysis and also pathways that reduce the availability of substrates necessary for aerobic metabolism [12, 13]. HIF-dependent upregulation of pyruvate dehydrogenase kinase 1 (PDK-1) inhibits pyruvate dehydrogenase (PDH) and the utilization of pyruvate to support aerobic metabolism [12, 13]. HIF-1α inhibition should decrease glycolysis in tumors and force energy production towards aerobic mitochondrial metabolism [9], thereby reducing lactate concentration but also increasing oxygen consumption and potentially making tumors more hypoxic [14]. There also is evidence to indicate that HIF-1α inhibition in the setting of hypoxia impairs the survival of cells that depend on glycolysis for energy production, leading to a process of hypoxia-induced cell death [15, 16].

With advancements in radiation physics there is increasing interest in delivering high-dose single-fraction hypofractionated radiation (HD-SFRT) to solid tumors using techniques such as stereotactic radiosurgery or brachytherapy [17]. Although HD-SFRT has shown promise in achieving improved local control, the biology of tumor response to high-dose radiation may differ from that of fractionated radiotherapy [18]. For example, previous studies have shown that the response to fractional radiation doses greater than 10 Gy is mediated in part by endothelial cell apoptosis and damage [19, 20]. It has also been suggested that tumor hypoxia may influence the response to HD-SFRT more than the response to fractionated radiotherapy because the important benefit of reoxygenation between fractions is lost [18]. Preclinical and modeling studies have shown that tumor hypoxia can induce a significant degree of resistance to HD-SFRT [21, 22].

Here, we investigate whether inhibiting HIF-1α in solid tumors will compromise the ability of cells to undergo glycolysis, especially under hypoxic conditions, force metabolism towards oxidative phosphorylation, increase oxygen consumption and consequently lead to cell death and increased effectiveness of HD-SFRT.

## Methods

### Experimental design

Parallel studies of tumor metabolism and radiation-induced growth delays were conducted in different groups of animals with or without HIF-1α inhibition. In one group, mice with HIF-1α wild type (HIF-WT) or HIF-1α knockdown (HIF-KD) tumors were either under air-breathing conditions or exposed to hypoxia and then sacrificed for tumor analysis. In the other group, mice with HIF-WT or HIF-1α KD tumors were exposed to the same conditions (air-breathing or hypoxia), irradiated under normoxia after the exposure, and followed to evaluate tumor growth delay. Tumor hypoxia and metabolism measured in the first group were compared to growth delay measured in the second group.

### Mice, tumor cell lines and HIF-1α inhibition

Experiments were performed using the ME180 (human cervix cancer, ATCC, HTB-33) and FaDu (human head and neck, ATCC, HTB-43) tumor cell lines. ME180 cells were grown as monolayers in plastic tissue culture flasks using α-MEM medium supplemented with 10% fetal bovine serum. FaDu cells were grown in a similar protocol except with MEM-F15 medium with 10% fetal bovine serum. Cells were maintained in a humidified tissue culture incubator under 5% CO_2_.

For ME180 cells, HIF-1α KD was achieved through a doxycycline-inducible HIF-1 shRNA. This system was developed using the Flp-In T-Rex Core Kit from Invitrogen (Breda, NL, USA) according to the manufacturer’s recommendations. To induce HIF-1α KD, 5 g/L doxycycline was added to the drinking water of the mice when tumor growth was observed (approximately 5 mm diameter) for 5 days prior to analysis. To achieve HIF-1α KD in FaDu cells, lentiviral transfection of a HIF-1α shRNA was performed. HIF-WT FaDu cells were transfected with the empty vector. In both cell lines, HIF-1α KD was confirmed with western blot analysis in vitro and RT-PCR was employed to verify KD in vivo.

Intramuscular (i.m.) tumors were generated by injection of 1 × 10^5^ cells in a 50 μl volume of the appropriate media into the left gastrocnemius muscle of syngeneic 8–12 week old nu/nu female mice (NU-Fox1^nu^, Charles River Laboratories, Senneville, QC, Canada). Growth delay experiments in ME180 tumors were carried out using 6–8 week old NRG mice (NOD-Rag1^null^ IL2rg^null^, Ontario Cancer Institute, in-house breeding colony). The animals had access to food and water ad libitum.

### Western blot

Protein lysates from ME180 and FaDu cells grown in vitro were collected as previously described and stored at --80 ºC [23]. Briefly cell lysates were isolated with RIPA buffer (20 min at 12000 rpm 4 °C). Protein concentrations were determined using a BCA protein assay (Pierce Biotechnology). Denatured proteins (40μg) were separated by SDS-PAGE 10% [*w*/*v*] gels, and transferred to nitrocellulose membranes (Amersham) using the Mini Trans-Blot System (BioRad). Membranes were incubated overnight at 4 °C with human anti-mouse HIF-1alpha (BD Bioscience 1:50) and with anti-rabbit Actin (Sigma; 1:2000) for equal protein loading. Blots were washed with PBS and incubated for 1 h at room temperature with fluorescent dye-labeled secondary antibodies. Protein detection and quantification was performed using the Odyssey Imaging System.

### qRT-PCR for CAIX

Total RNA was extracted using the RNeasy Mini Extraction kit (Qiagen, Valencia, CA, USA) from frozen tissue according to the manufacturer’s instructions. From 0.5 μg of DNase-treated total RNA, first-strand cDNA was reverse-transcribed using OmniScript (Qiagen, Valencia, CA, USA). For real-time PCR detection, cDNA (1/10) was mixed with primers (0.3 μM), ddH20 and SYBR Green Master Mix (Applied Biosystems, Carlsbad, CA, USA) with a well volume of 20 μl. Human CAIX primer sequences (Forward: 5`-CCTCAAGAACCCCAGAATAATGC-3`; Reverse: 5`-CCTCCATAGCGCCAATGACT-3`) were synthesized by Invitrogen. The real-time PCR protocol consisted of 40 cycles at 50 °C for 2 min, 95 °C for 10 min, 95 °C for 15 s and 60 °C for 1 min. The reactions were run and analyzed on an ABI 7900 Sequence Detector (Applied Biosystems, Carlsbad, CA, USA). Human L32, YWAZ, and HPRT were used as endogenous controls for normalization. Samples were run in triplicate to obtain the corresponding threshold cycle values, which were used as a direct quantitative measurement of gene expression level.

### In vitro oxygen consumption and Glycolysis

In vitro oxygen consumption rate (OCR) and Extracellular acidification rate (ECAR) in ME180 and FaDu cells was determined using the Seahorse XF96 Extracellular Flux Analyzer, as previously described [24]. Briefly, 20,000 cells were seeded in XF^e^96 microplate with complete media at 21% O_2_. Post 2 h of seeding, one plate was transferred to 0.2% O_2_ hypoxic chamber for 24 h incubation. The second plate was continued for additional 21 h at 21% O_2_ and then incubated at 0.2% O_2_ for 3 h. The third plate was maintained in normoxia for a total of 26 h. After incubation the complete medium was removed and replaced with with 150 μl bicarbonate-free medium in a CO_2_ free incubator for 1 h before analysis. Basal and maximal mitochondrial respiration (OCR) was measured in the presence of ATP synthase inhibitor, Oligomycin (1 μmol/L) and mitochondrial uncoupler, FCCP (0.5 μmol/L). Extracellular acidification rate (ECAR) was measured as a surrogate for lactate production and glycolysis. Data was normalized by cell number per well and quantified using CyQUANT NF cell proliferation assay kit (#C7026, ThermoFisher Scientific).

### In vivo hypoxia exposure

Mice were placed in an environmental chamber and exposed to a continuous flow of humidified 7% O_2_ and balanced N_2_ gas mixture for 3 h. For tumor bioluminescence and histological analysis, mice were immediately sacrificed upon removal from the environmental chamber after 3 h exposure. For growth delay experiments, the mice in the radiation group were removed from the environmental chamber and placed immediately in the irradiator. Radiation was delivered while the animals were breathing room air.

### Radiation treatment

Tumor bearing mice (i.m. tumors 8–10 mm diameter) were administered a single dose of 20 Gy, at a dose rate of 3.17 Gy/min, using a parallel opposed technique with a 225 KVp irradiator (XRad 225 Cx) [25]. A specially designed lucite jig was used to ensure targeted radiation of the tumor-bearing limb only.

### Tumor growth delay

Tumor growth was monitored by measuring the external leg diameter every 2–3 days. Measurements were performed blinded to the treatment group. The leg diameter was converted into weight in grams through a standard curve (leg diameter vs weight) generated by our group based on excising and weighing previous intramuscular leg tumour models in our lab. Standard deviations are indicated. The mice were sacrificed when the tumors reached a diameter of 15 mm. Growth curves were normalized to account for variations in the initial sizes. For each group, the median time for tumors to double (FaDu) or reach 2.5 times the original size (ME180) was determined. These endpoints were chosen based on the average final tumor sizes of the two tumor types.

### Analysis of Microvessel density (CD31) and proliferation (Ki-67)

Tumor sections were labeled for fluorescence microscopy, using primary antibodies against CD31 (Santa Cruz Biotechnology, Santa Cruz, CA) and Ki-67 clone sp6 from Neomarkers (Lab Vision, Freemont, CA). Secondary Cy5-conjugated anti-rat or anti-rabbit antibodies were used for indirect immunofluorescence staining (Jackson Laboratories, Bar Harbour, ME). Secondary antibodies were used alone to control for nonspecific background.

Entire immunofluorescence-stained sections were imaged at 0.5 μm resolution, using a laser-scanning whole-slide imager (TISSUEscope; Huron Industries, Waterloo, ON, Canada), and composite images of regions of interest were imaged at higher resolution (20X), using a conventional fluorescence microscope and scanning stage (BX50; Olympus Corporation). Uncompressed TIFF images (8-bit) were acquired for analysis.

H&E-stained images of adjacent tissue sections were reviewed to generate viable tumor masks. The fluorescence intensity in viable tumor areas was quantitated using Image-Pro Plus 6.1.0 (Media Cybernetics, Bethesda, MD). The Immunofluorescence intensity was visually inspected and was represented by intensities above the 75th percentile. The integrated optical density and fractional labeled area were measured in viable tumor areas using the 75th percentile threshold. Relative protein abundance was defined as the product of the integrated optical density and fractional labeled area.

### Analysis of tumor hypoxia

Analysis of hypoxia was carried out using the hypoxia marker EF5 [2-(2-nitro-1*H*-imidazole-1-yl)*N*-(2,2,3,3,3-pentafluoropropyl) acetamide] using a published protocol [26]. Tumor bearing animals were injected with EF5 at 10 mg/kg 3 h prior to tumor excision. Once excised, tumors were snap frozen in liquid nitrogen. Slides were then processed according to standard immunohistochemical protocols. The primary antibody used for EF5 was the biotinylated antibody ELK 3.51 at a concentration of 1 mg/ml.

The entire stained sections were analyzed by a board-certified veterinary pathologist. EF5 immunohistochemical staining was scored based on the percentage of staining in viable tumor tissue after excluding regions of necrosis. The analysis for viable regions was as according to pathology principles. Necrosis was identified based on changes in cell morphology such as increased eosinophilic staining (on HE slides); shrinkage, fragmentation and loss of nuclei; dissolution of cell membranes - all resulting in a loss of cellular and nuclear definition.

### Bioluminescence microscopy of ATP and lactate

An instrument and protocols for bioluminescence microscopy of tumor sections to map lactate and ATP was developed in-house based on the technique described by Mueller-Klieser and colleagues in Germany [27]. Briefly, tumor cryosections were placed in contact with an enzyme solution containing luciferase, a light-emitting enzyme obtained from firefly or bacteria. The lactate reaction depends on bacterial luciferase and the production of NADPH. For ATP, the bioluminescence reaction is achieved through a solution containing firefly luciferase. Multiple tumor cryosections (2–4), spaced either 32 or 77 μm apart, were measured to account for heterogeneity. The closest section used to evaluate ATP or lactate was 26 μm from a parallel EF5 section. Non-tumorous and necrotic regions were excluded from the analysis using parallel H&E sections that were reviewed by a pathologist to ensure consistency. Bright field images of the ATP and lactate sections were obtained to align the bioluminescence images with the H&E and EF5 sections.

To calibrate the concentration of lactate and ATP with the bioluminescence intensity, known concentrations of these metabolites were dissolved in 0.1 M PBS, mixed with OCT Tissue Tek, frozen and fixed to a slide for bioluminescence microscopy. These were used to generate a standard curve of bioluminescence signal intensity versus metabolite concentration.

**Fig. 1:**
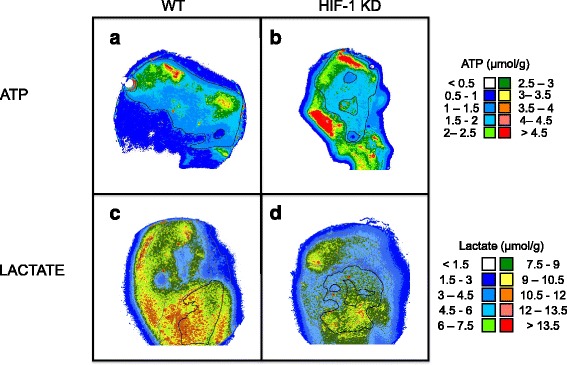
Representative bioluminescence images of ATP in ME180 sections (**a**,**b**) and lactate in FaDu sections (**c**,**d**), for both HIF-WT (*WT*) (**a**, **c**) and HIF-1α KD (*HIF-1 KD*) (**b**, **d**) tumors under hypoxic conditions (7% oxygen for 3 h). The viable tumor areas (*black outline*) from the corresponding H&E sections were mapped onto these bioluminescence images for quantification

The mean intensity within viable tumor regions was then calculated to obtain ATP and lactate concentration. Figure 1 shows examples of ATP and lactate images from a ME180 and FaDu tumor exposed to hypoxia before and after HIF-1α KD.

### Statistical analysis

Mean values of ATP, lactate, EF5, CD31 and Ki-67 positivity were obtained from groups of 5–11 tumors. Standard error of the mean for each are indicated. Differences between groups were evaluated using the two-tailed Mann-Whitney non-parametric test. Differences in the time for irradiated HIF-WT and HIF-1α KD tumors to regrow to a pre-defined size were also assessed using the Mann-Whitney test.

## Results

### HIF-1α knockdown

**Fig. 2:**
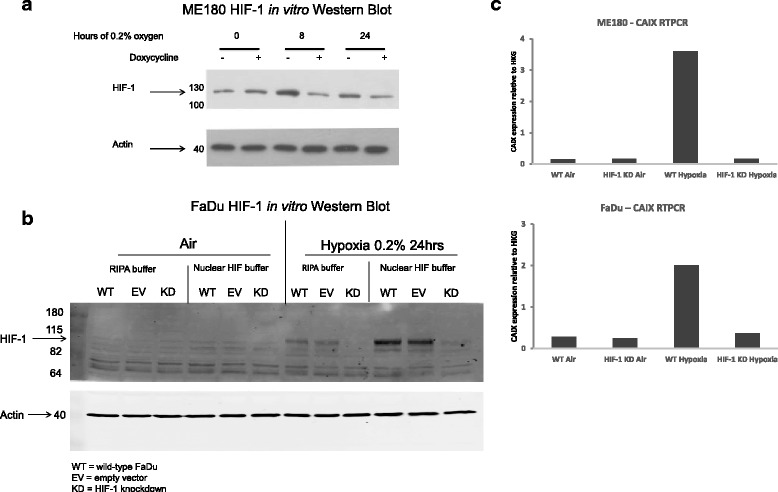
**a** and **b** show Western Blot analysis of HIF-1α protein levels from ME180 HIF-WT (*DOX-*) and HIF-1 KD (*DOX+*) cells and FaDu HIF-WT (*WT*), empty vector control (EV) HIF-1 KD (*HIF-1 KD*) cells in air (0 h) and 0.2% oxygen (8, 24 h ME180; 24 h FaDu). **c** shows RTPCR for CAIX, which confirms downstream inhibition of CAIX mRNA transcription following HIF-1 protein knockdown (HKG, housekeeping genes)

HIF-1α KD was observed in both cell types, FaDu and ME180 (Fig. 2a and b). Low levels of HIF-1α protein were detectable under 21% O_2_ conditions in both HIF-WT and HIF-1α KD FaDu and ME180 tumors. HIF-1α protein was induced by hypoxic exposure, and this induction was attenuated in both the ME180 and FaDu KD cells (Fig. 2 and b).

RTPCR analysis of the HIF-1 target gene CAIX verified the knockdown of HIF-1 transcriptional activity in these cell lines (Fig. 2c). Similar to HIF-1α protein, low levels of CAIX mRNA were present in 21% O_2_. After hypoxia, CAIX gene expression increased significantly in HIF-WT cells, but did not change in HIF-KD cells, highlighting the HIF-dependence of CAIX expression and the functional effect of HIF knockdown in these lines.

### In vitro oxygen consumption

**Fig. 3:**
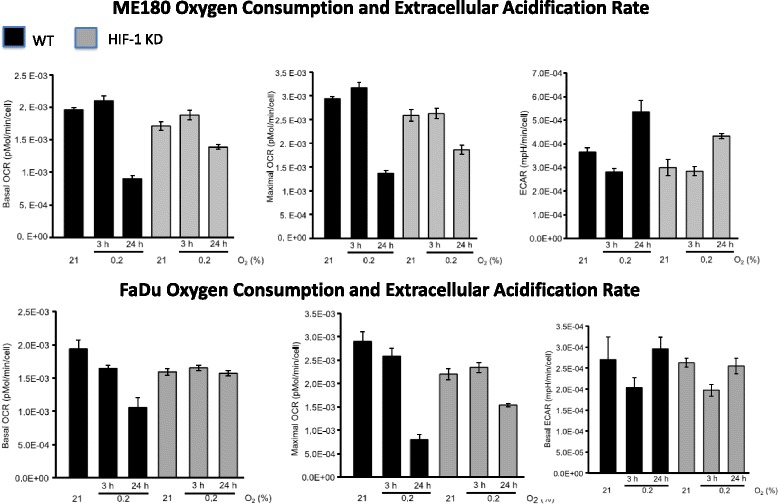
In vitro oxygen consumption rates (OCR) and extracellular acidification rates (ECAR) in HIF-WT and HIF-1α KD cells: (*top*) ME180, (*bottom*) FaDu

It has been previously shown that 12–24 h h of hypoxia causes a reduction in oxygen consumption that persists for up to 12 h upon reoxygenation [14]. To examine this phenomenon in these cell lines, basal and maximal oxygen consumption rates were measured after exposing ME180 and FaDu cells to 24 h of 0.2% O_2_, 1–2 h after reoxygenation (Fig. 3). Both basal and maximal oxygen consumption was significantly lower in ME180 and FaDu HIF-WT cells exposed to hypoxia (0.2%) compared to HIF-WT cells maintained in normoxic conditions, in keeping with a shift from aerobic to anaerobic metabolism. HIF-KD blocked some of this hypoxic effect in both cell lines. Oxygen consumption was significantly higher in HIF-KD cells compared to HIF-WT cells exposed to hypoxia (0.2%). Lactate production (ECAR) was significantly lower in both ME180 and FaDu HIF-KD cells exposed to 24 h of 0.2% O_2_, compared to HIF-WT cells exposed to the same hypoxic conditions.

These effects appear to require extended hypoxic exposure, as under normoxia and short-term hypoxia (0.2% O_2_, 3 h), there were no significant differences in OCR or ECAR between HIF-1α KD and HIF-WT cells upon reoxygenation in either the ME180 or FaDu lines.

### Hypoxic fraction

**Fig. 4:**
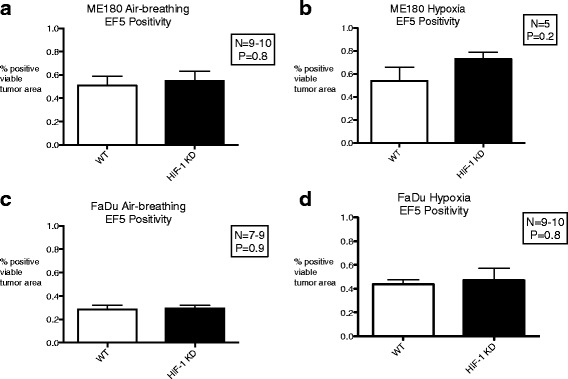
Mean EF5 scores in ME180 and FaDu tumors under air-breathing conditions (**a**, **c**) and during 3 h of exposure to 7% O_2_ breathing conditions (**b**, **d**). Error bars represent standard error of the mean

ME180 HIF-WT tumors had significantly higher levels of hypoxia than FaDU HIF-WT tumors under air breathing conditions (EF5 HF 0.51 ± 0.08 vs. 0.28 ± 0.03, *p* = 0.009), as shown in Fig. 4. Short-term exposure of HIF-WT tumors to a low O_2_ environment (7% O_2_ for 3 h) had no effect on tumor hypoxia in ME180 (EF5 HF 0.51 ± 0.08 vs. 0.54 ± 0.12, *p* = 0.6) but significantly increased the hypoxic fraction in FaDu HIF-WT tumors (EF5 HF 0.28 ± 0.03 vs. 0.44 ± 0.03, *p* = 0.007) relative to air breathing conditions.

There was no difference in tumor hypoxia (ME180: EF5 HF 0.55 ± 0.08vs. 0.51 ± 0.08, *p* = 0.5; FaDu: EF5 HF 0.29 ± 0.03 vs. 0.28 ± 0.03, *p* = 0.9) between HIF-KD and HIF-WT ME180 or FaDu tumors under air breathing conditions. Following short-term hypoxia exposure, there was a trend towards greater tumor hypoxia in ME180 HIF-KD tumors compared to HIF-WT (EF5 HF 0.73 ± 0.06 vs. 0.54 ± 0.12, *p* = 0.2) but no difference in FaDu tumors (EF5 HF 0.47 ± 0.03 vs. 0.44 ± 0.1, *p* = 0.3).

There were no changes in microvessel density (CD31) or proliferation (Ki-67) in either tumor model in response to hypoxia or HIF1 inhibition (data not shown). These factors were assessed because they could influence oxygen supply and consumption respectively.

### Lactate and ATP

**Fig. 5:**
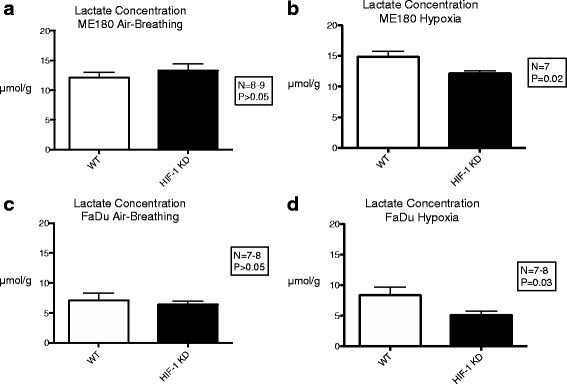
Mean lactate concentration measured using bioluminescence imaging in ME180 and FaDu tumors under air-breathing conditions (**a**, **c**) and after exposure to 3 h of 7% O_2_ breathing conditions (**b**, **d**). Error bars represent standard error of the mean

The mean lactate concentration in HIF-WT tumors as measured using bioluminescence microscopy was significantly higher in ME180 than in FaDU (12.1 ± 0.86 vs. 7.1 ± 1.2 umol/g, *p* = 0.008) under air breathing conditions (Fig. 5). Short-term exposure of HIF-WT ME180 tumors to a low oxygen environment significantly increased lactate concentration (14.9 ± 0.89 vs. 12.1 ± 0.86 umol/g, *p* = 0.05) relative to air breathing but had no effect in FaDu HIF-WT tumors (8.3 ± 1.3 vs. 7.1 ± 1.2 umol/g, *p* = 0.7).

There was no difference in lactate concentration (ME180: 13.3 ± 1.1 vs. 12.1 ± 0.86, *p* = 0.5 Mann-Whitney; FaDu: 6.4 ± 0.6 vs. 7.1 ± 1.2, *p* = 0.4) between HIF-KD and HIF-WT ME180 or FaDu tumors under air breathing conditions. However, following short-term hypoxia exposure, there were significantly lower tumor lactate concentrations in both the ME180 (12.1 ± 0.43 vs. 14.9 ± 0.89, *p* = 0.02 Mann-Whitney) and FaDu (5.1 ± 0.67 vs. 8.3 ± 1.3, *p* = 0.02 Mann-Whitney) HIF-KD tumors relative to their HIF-WT counterparts.

There were no significant differences in mean ATP levels as measured by bioluminescent microscopy between HIF-WT and HIF-KD tumors for either ME180 or FaDu tumors, regardless of hypoxic exposure (data not shown).

### Radiation treatment response – Tumor growth delay

Tumour weights at the time of radiation varied in ME180 tumors due to the differences in growth during doxycycline treatment (mean = 0.59 ± 0.12 g). FaDu tumor weights (mean = 0.31 ± 0.05 g) were more consistent as compared to ME180 as there was no need for doxycycline treatment due to lentirviral transfection of HIF-KD.

**Fig. 6:**
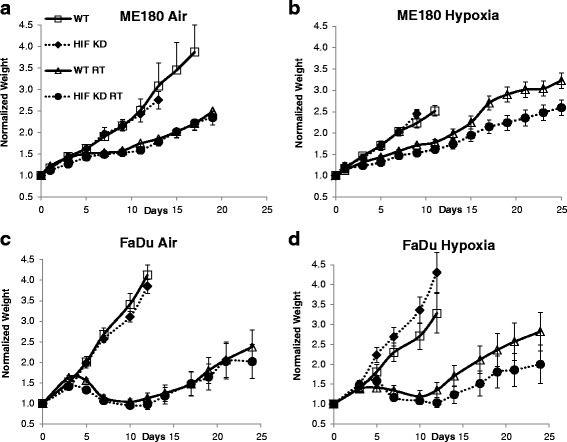
Growth curves for ME180 and FaDu tumors each with and without high-dose radiation (20Gy single fraction) administered on day 0 under air-breathing conditions (**a**, **c**) or immediately following exposure to 3 h of 7% O2 breathing (**b**, **d**) conditions (*n* = 4–10 mice per group). The tumor weight in each animal was normalized to its weight at the start of the experiment

Unirradiated ME180 and FaDu HIF-1α KD tumors grew at the same rates as the corresponding HIF-WT tumors under both normoxic and hypoxic conditions, with no effect on the health or activity of the mice (Fig. 6). Unirradiated HIF-WT ME180 tumors grew more slowly than HIF-WT FaDu tumors and were less radioresponsive. Of note, the ME180 HIF-WT tumors were found to be more hypoxic and to have higher lactate concentrations than FaDu tumors (Fig. 4), which may contribute to their lower radiosensitivity (Fig. 4).

For both ME180 and FaDu tumors irradiated under air-breathing conditions, HIF-1α KD had no significant effect on the tumor regrowth. However, for tumors exposed to hypoxia and then irradiated after reoxygenation, HIF-1α KD resulted in a significant increase in growth delay (*p* = 0.038 ME180, *p* = 0.049 FaDu). Also, as illustrated in Additional file 1: Figure S1, in the ME-180 model, HIF-WT tumors regrew more quickly after exposure to hypoxia than all other irradiated groups (HIF-WT air, HIF-KD air, HIF-KD hypoxia).

## Discussion

Tumor cells are adapted to survive in hypoxic and acidic microenvironments, in part through a switch to anaerobic metabolism with increased glucose consumption and increased lactate production [9]. High lactate levels have been measured in several human cancers and associated with poor patient survival [3, 4]. Pre-clinical studies have suggested a correlation between lactate levels and radioresistance [5]. It is not known if lactate influences radiation response directly or is a surrogate for other and cellular molecular processes also associated with anaerobic metabolism. Regardless, the cumulative evidence suggests that targeting anaerobic metabolism in tumors is an attractive therapeutic strategy to improve the effectiveness of radiotherapy. The approach used in this study was to induce transient hypoxic stress in tumors after long-term inhibition of HIF-1α, a key regulator of glycolytic enzymes and the availability of substrates for aerobic mitochondrial metabolism [9, 12], and evaluate the impact of both maneuvers on hypoxia, lactate levels and response to HD-SFRT.

In general, there were potentially important biological differences in the response to hypoxic stress between HIF-WT and HIF-KD tumors. These findings suggest that short-term exposure to a low oxygen environment (7% O_2_ for 3 h) increased tumor hypoxia during the exposure, consistent with previous reports [28]. These previous data suggest that reoxygenation likely occurred rapidly during the short interval between removing the animal from the environmental chamber and sacrifice. Lactate levels following hypoxia exposure were significantly higher in HIF-WT than in HIF-KD tumors, indicating that the latter have less capacity to rapidly adapt to hypoxic stress by increasing anaerobic metabolism. In addition, tumors with intact HIF-1α subjected to HD-SFRT immediately following hypoxia exposure were less responsive to treatment than tumors without functional HIF-1α, and tumors irradiated under air breathing conditions regardless of HIF-1α status (Fig. 6 and Additional file 1: Figure S1). This implies a potentially important interactive effect between tumor hypoxia and HIF-1α inhibition on radiation treatment response.

HIF-1α inhibition altered tumor metabolism in mice exposed to a low oxygen environment (7% O_2_ for 3 h) but had minimal effect on tumors in air-breathing animals. Low oxygen breathing as used in this study induced hypoxic stress in the tumors but probably was too short to significantly alter HIF-dependent (HIF-WT tumors) or independent gene expression. Our findings, therefore, are likely to reflect the different inherent responses of the HIF-WT and HIF-KD tumors to acute hypoxic stress. In the presence of normal HIF with high expression of glycolytic genes, cells were able to adapt to short-term hypoxia by immediately increasing anaerobic metabolism, reducing their oxygen dependence and increasing lactate production. In the absence of HIF, this response was blunted; cells continued to rely on oxygen and may have been more likely to die because of insufficient oxygen to meet continuing demand. It is noted that no significant effect on metabolism was seen in vitro with exposure to 3 h of hypoxia (effect was seen in 24 h). However this may not be comparable to the in vivo scenario where there are oxygen gradients and the effect of externally applied hypoxia is a shift in a subpopulation of the cells further along this gradient.

An important result of this study is enhanced radiation treatment response following the induction of short-term hypoxic stress in tumors subjected to long-term HIF inhibition. In animals exposed to a low oxygen environment immediately prior to HD-SFRT, HIF-WT tumor progressed significantly more rapidly than HIF-KD tumors (Fig. 6 and Additional file 1: Figure S1). This effect was not seen under normal air breathing conditions. In fact, the growth curves for animals in the air breathing arms (regardless of HIF status) and the hypoxic, HIF-KD arm were indistinguishable. There are several possible explanations for this. Given the observed changes in tumor hypoxia and lactate production, we hypothesize that this is due in part to complementary cell killing of radiosensitive versus hypoxic and radioresistant cell populations. HIF-WT cells were better able to rapidly adapt to acute hypoxic stress by switching to anaerobic metabolism, making them less dependent on oxygen for continued survival. In contrast, a proportion of the viable HIF-KD cells initially at low oxygen concentrations, having little capacity to rapidly modulate metabolism, were shifted to even lower oxygen levels incompatible with continued survival. HD-SFRT immediately after induction of hypoxic stress and reoxygenation selectively depleted the viable, oxic cell population. Tumor regrowth was then dominated by the viability of the more hypoxic cells, which manifested as differences in growth delay between HIF-WT and HIF-KD tumors. Similar findings have been reported with combinations of HD-SFRT and hypoxic cell cytotoxic drugs [29, 30] This mechanism could have potential as a clinical strategy by combining glycolytic inhibitors such as 2-DG and 3-BrPA with HD-SFRT for treatment of hypoxic tumors.

It is possible that other mechanisms may have contributed to the rapid regrowth of HIF-WT tumors following the induction of acute hypoxic stress, reoxygenation and HD-SFRT, and the relative protective effect of HIF inhibition. HIF-1α upregulation by hypoxia and reactive oxygen species (ROS) generated during reoxygenation can antagonize the cytotoxic effects of radiotherapy independent of metabolic status by directly altering cell survival and cell death signaling and/or indirectly by promoting endothelial cell survival and vasculogenesis [11, 31, 32]. In our experiments, the hypoxic mice were irradiated under air-breathing conditions as soon as possible after removal from the environment chamber, although there was a brief period of tumor reoxygenation that might have contributed to increased ROS production. This would be expected to have minimal impact on HIF-1α levels in the HIF-1α KD tumors but to promote HIF-1α stabilization and impaired treatment response in the HIF-WT tumors. An alternative explanation more directly related to a shift from aerobic to anaerobic metabolism is reduced scavenging of radiation-induced ROS by pyruvate and lactate in HIF-1α KD tumors [5, 7, 33]. Given the diverse effects of HIF-1, there are other mechanisms that may affect tumor growth in these systems. However, CD31 and Ki-67 staining were not altered in HIF-KD tumors, indicating that that vascularity and cell division rates were not detectably changed. Finally, future experiments should also examine the contribution of HIF-2 in addition to HIF-1 in mediating these effects, as both of these hypoxia sensing transcription factors may play a role in the observed effects, and may be viable targets for therapeutic intervention.

We also note that the short-term 7% O_2_ exposure did not have a significant effect on increasing EF5. This may be partly explained by the predominant effects of chronic hypoxia on the growing xenograft tumors, regardless of exposed air conditions. Any change of exposed oxygen may have had small effects as compared to the inherent chronic hypoxia from the irregular blood supply to the tumour. Also, we had designed this experiment based on the Cairns publication from our lab, where a decrease in %O_2_ in the exposed air resulted in a rapid decrease in pO_2_ of the mice leg tumors as measured by oxygen sensor probe measurements [28]. However, in our experiment we measured hypoxia with immunohistochemistry EF5 staining, which may not have represented this effect to the same degree. The goal of this applied oxygen exposure was to target the marginally hypoxic cells near the diffusion limit of oxygen. It is hypothesized that this will shift these cells to even lower oxygen levels where the adaptive, biological consequences of hypoxia become more evident (ie changes in lactate, radiation response). Whether this shift is measureable as an increase in global EF5 binding will depend on the degree of hypoxia achieved (relative to EF5 binding dynamics) and the percentage of the total tumor affected. It is possible that this short exposure may not been sufficient to influence the EF5 staining in a significant area of the tumours. Furthermore, EF5 was measured semi-quantitatively with IHC methods and the marginally hypoxic cells may not have been captured in the analysis. Also, activation of HIF-1 can occur at O_2_ levels below 10–15 mmHg, whereas maximal binding of EF5 occurs at lower O_2_ levels on the order of 1–2 mmHg or less [34]. The results of our study suggest that the effects of hypoxia exposure were to increase the proportion of mildly or moderately hypoxic cells but not the proportion of severely hypoxic or anoxic cells. The changes were sufficient to activate HIF and influence radiation response but may not have been sufficient to significantly change EF5 binding.

The two cell line models described in this study employ different methods for knocking down HIF in order to assess constitutive inhibition prior to tumor implantation (FaDu) and inducible inactivation of established tumors (ME-180). These complimentary approaches may have different impacts on the tumor microenvironment and metabolism downstream of HIF. Therefore, the data obtained for each model should be compared to the appropriate control group. Results that are consistent across both systems are likely to indicate robust effects that may not depend on the timing of HIF inhibition.

Also, NRG mice were used in the ME180 growth delay experiment as they became recently available in our lab for the study. Since they were more-immune-deprived and there is less concern of NK cell activity than in nude mice we opted to use them for the growth ME180 delay experiments (FaDu growth delay were already completed with nude mice). There may be a confounding factor as the ME180 metabolic experiments were performed with nude mice. Finally, in order to confirm the extent of inhibition of HIF1 activity by the HIF-1 knockdown targeting strategies in vivo, the expression of a canonical HIF-1 target gene was measured (CAIX). The response of CAIX expression to a hypoxic stimulus was abrogated in both lines. However, the relative induction of CAIX in the two lines differed somewhat, and may indicate a cell line dependent but HIF1 independent regulation of CAIX in these two cell lines.

## Conclusions

### Perspectives

Hypofractionated radiotherapy schedules are assuming greater prominence in clinical practice with the available of more robust external beam image guidance and tumor targeting capability. High-dose rate brachytherapy for prostate or cervical cancer capitalizes on the steep dose gradients inherent in these techniques to allow large fractional doses to be safely delivered. The response of tumors to a small number of large radiation fractions is likely to be more strongly dependent on hypoxia than the response to conventionally fractionated regimens because there is less opportunity for reoxygenation [18]. Thus, the results of this study may be directly relevant to clinical practice. They suggest that combining hypofractionated radiotherapy with metabolic reprogramming to inhibit anaerobic metabolism may improve patient outcomes through complementary killing of radiosensitive and hypoxic, radioresistant cell populations. The study also highlights the importance of including relevant biomarkers of metabolism in future pre-clinical and clinical radiation treatment studies. The bioluminescence approach used here to measure ATP and lactate concentration is readily applied in the clinic [3, 4], with the major requirement being the need to snap-freeze biopsies within a few seconds of acquisition. Evolving metabolic imaging techniques, including hyper-polarized magnetic resonance spectroscopy, offer the promise of serially evaluating metabolism in patients during treatment in a minimally invasive manner [35].

## Additional file

Additional file 1: Figure S1. Growth curves for irradiated ME180 tumors from Fig. 6, presented together for ease of comparison. (PPTX 35 kb)

## Abbreviations

ECAR: Extracellular acidification rate
HD-SFRT: High-dose single-fraction radiotherapy
HIF-KD: HIF-1α knockdown
HIF-WT: HIF-1α wild type
OCR: Oxygen consumption rate
PDH: pyruvate dehydrogenase
PDK-1: pyruvate dehydrogenase kinase 1
