# Supplementary material for: Metabolic targeting of HIF-dependent glycolysis reduces lactate, increases oxygen consumption and enhances response to high-dose single-fraction radiotherapy in hypoxic solid tumors
Source: BMC Cancer. 2017 Jun 15;17:418. doi: 10.1186/s12885-017-3402-6 (PMC5473006; doi:10.1186/s12885-017-3402-6)
Supplement: Additional file 1: Figure S1. — Growth curves for irradiated ME180 tumors from Fig. 6, presented together for ease of comparison. (PPTX 35 kb) [file 12885_2017_3402_MOESM1_ESM.pptx]

## Slide 1
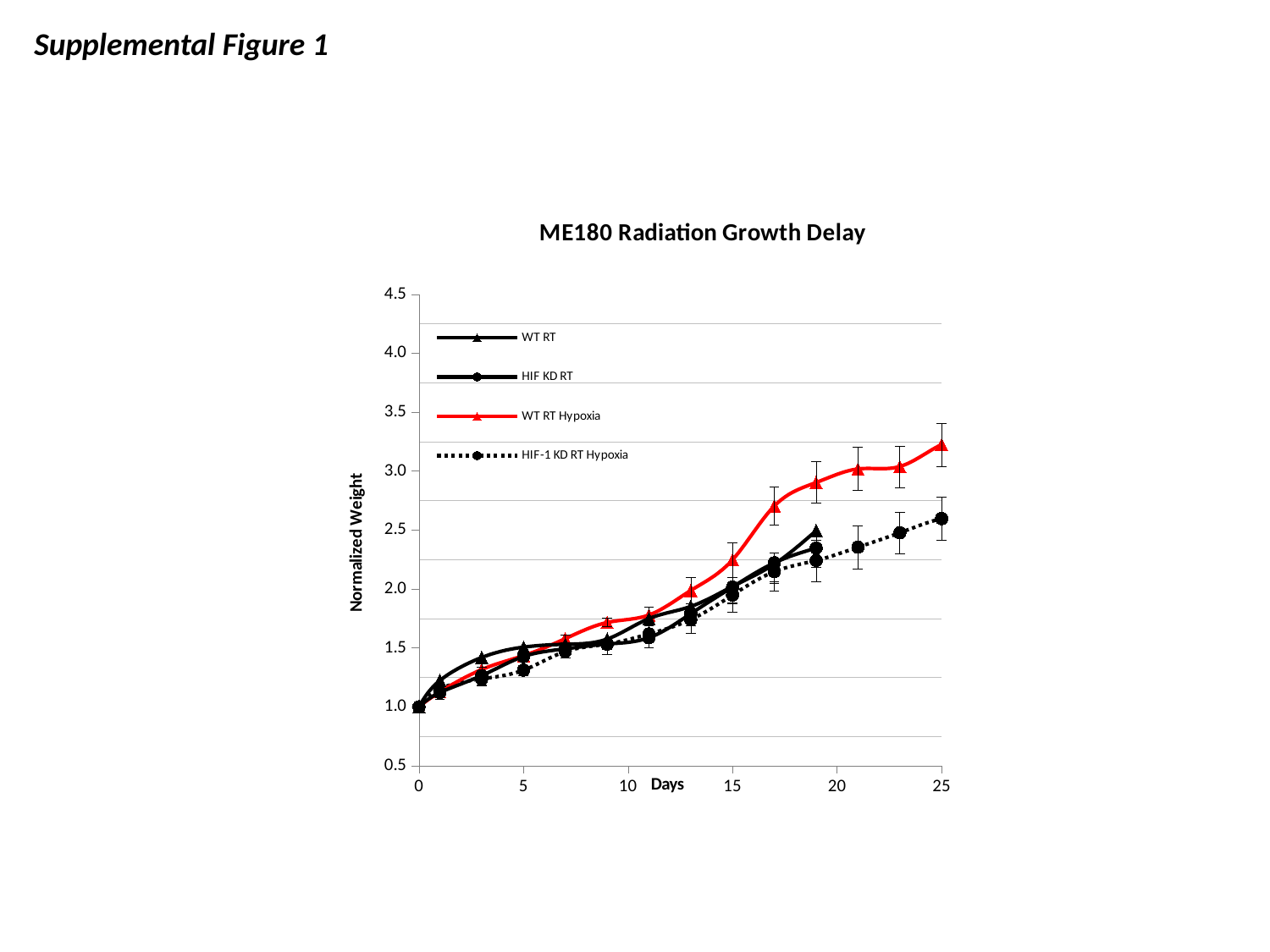

Supplemental Figure 1
### Chart: ME180 Radiation Growth Delay
| Category | WT RT | HIF KD RT | WT RT Hypoxia | HIF-1 KD RT Hypoxia |
|---|---|---|---|---|
